# Model selection for survival individualized treatment rules using the jackknife estimator

**DOI:** 10.1186/s12874-022-01811-6

**Published:** 2022-12-22

**Authors:** Gilson D. Honvoh, Hunyong Cho, Michael R. Kosorok

**Affiliations:** 1 Department of Biostatistics, University of North Carolina at Chapel Hill, Chapel Hill, NC USA; 2 Department of Physical Medicine & Rehabilitation, School of Medicine, University of North Carolina at Chapel Hill, Chapel Hill, NC USA

**Keywords:** Precision medicine, Individualized treatment rules, Right-censoring, Jackknife, Leave-one-out-cross-validation, Inverse probability of censoring weighting

## Abstract

**Background:**

Precision medicine is an emerging field that involves the selection of treatments based on patients’ individual prognostic data. It is formalized through the identification of individualized treatment rules (ITRs) that maximize a clinical outcome. When the type of outcome is time-to-event, the correct handling of censoring is crucial for estimating reliable optimal ITRs.

**Methods:**

We propose a jackknife estimator of the value function to allow for right-censored data for a binary treatment. The jackknife estimator or leave-one-out-cross-validation approach can be used to estimate the value function and select optimal ITRs using existing machine learning methods. We address the issue of censoring in survival data by introducing an inverse probability of censoring weighted (IPCW) adjustment in the expression of the jackknife estimator of the value function. In this paper, we estimate the optimal ITR by using random survival forest (RSF) and Cox proportional hazards model (COX). We use a Z-test to compare the optimal ITRs learned by RSF and COX with the zero-order model (or one-size-fits-all). Through simulation studies, we investigate the asymptotic properties and the performance of our proposed estimator under different censoring rates. We illustrate our proposed method on a phase III clinical trial of non-small cell lung cancer data.

**Results:**

Our simulations show that COX outperforms RSF for small sample sizes. As sample sizes increase, the performance of RSF improves, in particular when the expected log failure time is not linear in the covariates. The estimator is fairly normally distributed across different combinations of simulation scenarios and censoring rates. When applied to a non-small-cell lung cancer data set, our method determines the zero-order model (ZOM) as the best performing model. This finding highlights the possibility that tailoring may not be needed for this cancer data set.

**Conclusion:**

The jackknife approach for estimating the value function in the presence of right-censored data shows satisfactory performance when there is small to moderate censoring. Winsorizing the upper and lower percentiles of the estimated survival weights for computing the IPCWs stabilizes the estimator.

**Supplementary Information:**

The online version contains supplementary material available at 10.1186/s12874-022-01811-6.

## Introduction

Precision medicine aims to inform clinical decisions by tailoring treatments to the characteristics of each patient. By using individual information—such as clinical history, lab results, demographics and social and economic characteristics—precision medicine takes advantage of heterogeneity to provide treatment recommendations to each patient in a data-driven way [1]. Approaches for estimating an optimal treatment rule have been developed using machine learning techniques [2–6]. An optimal treatment rule is found among a class of all possible treatment rules that maximizes the “value”, i.e., the expected reward of a potential outcome when the rule is applied to future patients [2]. Since the value is used to evaluate the performance of a treatment rule, it is important to estimate its bias and standard error accurately. Such estimation is commonly done by cross-validation (CV). The jackknife estimator or leave-one-out-cross-validation (LOOCV) is a special case of CV where each individual in the sample is a fold, all but one fold are used for training and then the trained model is tested on the left-out fold. The procedure is repeated until all folds have been used as the test set once. The jackknife method requires very few assumptions and is approximately unbiased [7].

Jiang et al. [7, 8] used the jackknife estimator for determining the optimal individualized treatment rules for participants in the Intensive Diet and Exercise for Arthritis (IDEA) trial [9, 10]. For the three treatments for knee osteoarthritis available in the trial (exercise, dietary weight loss, and a combination of exercise and dietary weight loss) and seven outcomes of interest, the authors applied their proposed method to 27 different models, including penalized regression, kernel ridge regression, tree-based methods, list-based methods, and Bayesian additive regression trees. The method identified random forest as the optimal model for most outcomes, while list-based models were optimal for a few. The results supported the overall findings from the IDEA trial that the combined treatment intervention was optimal for most participants. However, one notable finding of the precision medicine approach was the evidence that, for certain outcomes, a subgroup of participants would benefit more from diet alone.

In biomedical research, right-censored time-to-event data are frequently collected because of dropouts or administrative censoring. In such settings where the interest is in a survival outcome, parametric and semi-parametric methods for estimating an individualized treatment rule have been developed to account for censoring [11–13]. Assuming independent or conditionally independent censoring, these methods generally perform well for a small to moderate proportion of censored observations. They are generally categorized as regression-based [14–16] or classification-based methods [11, 12]. Regression-based approaches model the mean outcome conditional on treatment assignment and patient data and their performance depends on the correct specification of the posited models. Classification-based approaches were developed to avoid issues of model misspecification. Within this second class of approaches, the optimal treatment rule is determined by the classifier that minimizes the expected weighted misclassification error. For both types of approaches, an important concern resides in finding tools for evaluating the performance of an ITR. In this paper, we extend the jackknife method proposed by Jiang et al. [7] for a binary treatment and a right-censored survival outcome. Our extension of the jackknife method uses the inverse probability of censoring weighting (IPCW) to mitigate the bias induced by the censored observations. We show the consistency of the jackknife estimator for right-censored data and study its asymptotic properties through simulations.

## Methods

### The jackknife estimator of the value function

In this section, we introduce general notations and present some existing work relevant to this paper. Let $\{(\mathbf {X}_{\mathbf {i}}, A_i, Y_i )\}_{i=1}^n$, be independent and identically distributed triples $({\mathbf {X}}, A, Y )$ for *n* patients. We define $\mathbf {X} \in \mathcal {X} \subseteq \mathbb {R}^p$ as a vector of baseline patient characteristics, $A \in \{0, 1 \}$ as the binary treatment indicator, and $Y \in \mathbb {R}$ as a scalar outcome, with higher values of *Y* representing more favorable outcomes. Precision medicine looks to maximize the expected reward of a potential outcome under a treatment rule. The treatment rule maps the patient-level covariates $\mathcal {X}$ to the binary treatment space $\{ 0, 1 \}$. In a single stage setting, the optimal treatment rule is selected among a class of all possible treatment rules $\mathcal {D}$ that maximizes the value of a potential outcome when applied to future patients [2]. The expected reward or value function is defined as:

$$\begin{aligned} V(d) = E^d (Y) = E\left[ Y \frac{1 \{ A=d (\mathbf {X})\}}{P(A|\mathbf {X})} \right] , \end{aligned}$$

where $P(A|\mathbf {X})$ is the propensity score, which is known in a randomized study or can be estimated with a logistic regression or some other model in an observational study. The optimal treatment rule is defined as $d^* = \arg \max _{d} V(d)$.

In the right-censored survival data setting, we consider $\tilde{T}$ as the true survival time and $T= min(\tilde{T}, \tau )$ as the $\tau$-truncated survival time where $\tau < \infty$ is the maximum follow-up time. Also, we assume censoring time *C* is independent of *T* given $({\mathbf {X}}, A)$ and denote the censoring indicator as $\delta =I(T \le C)$. When the interest is in maximizing the restricted mean survival time, the expression of the above value function then becomes

$$\begin{aligned} V(d) = E\left[ T \frac{1 \{ A=d (\mathbf {X})\}}{P(A|\mathbf {X})} \right] . \end{aligned}$$

The optimal treatments are estimated with regression-based or classification-based approaches. Regression-based approaches model the mean outcome conditional on treatment assignment and patient data [3, 17–19]. The performance of the regression-based approaches depend on the correct specification of the posited models. In addition, modeling treatment-covariate interactions can lead to a high-dimensional situation, further complicating the estimation procedures. Classification-based approaches were developed to avoid issues of model misspecification [4, 5, 20]. This alternative class of approaches relies on fewer modeling assumptions and the optimal treatment rule is estimated by minimizing the expected weighted misclassification error. The selected approach is then evaluated via cross-validation techniques for assessing the value function associated with the estimated treatment rule.

Jiang et al [7] proposed a LOOCV or jackknife estimator for the value function for continuous outcomes. The approach requires weak assumptions, namely that the data are independent and identically distributed. The proposed jackknife estimator is expressed as:

1 $$\begin{aligned} \widehat{V}^{jk} (\hat{d}_{n}) = \frac{ \sum _{i=1}^{n} Y_{i} \frac{1\left\{A_{i}=\hat{d}^{(-i)}_{n} (\mathbf {X}_{i})\right\}}{{P}(A_{i}|\mathbf {X}_{i})} }{ \sum _{i=1}^{n} \frac{1\left\{A_{i}=\hat{d}^{(-i)}_{n} (\mathbf {X}_{i})\right\}}{{P}(A_{i}|\mathbf {X}_{i})}} , \end{aligned}$$

where $\hat{d}^{(-i)}_{n}$ represents the treatment rule estimated from a training set of size *n* with the *i*-th observation left out and ${P}(A_{i}|\mathbf {X}_{i})$ is the propensity score of the test set *i*. The variance of the estimator in (1) is estimated by

$$\begin{aligned} \widehat{Var}\left[\widehat{V}^{jk} (\hat{d}_{n})\right] = \frac{1}{n (n-1)} \sum _{i=1}^{n} \left(R^{jk}_{i}\right)^2, \end{aligned}$$

where $R_i^{jk}= \frac{1}{\overline{W}_{n}} U_i - \frac{\overline{U}_{n}}{\overline{W}^2_{n}} W_i$, with $U_i = Y_{i} \frac{1\left\{A_{i}=\hat{d}^{(-i)}_{n} (\mathbf {X}_{i})\right\}}{{P}(A_{i}|\mathbf {X}_{i})}$, $W_i= \frac{1\left\{A_{i}=\hat{d}^{(-i)}_{n} (\mathbf {X}_{i})\right\}}{{P}(A_{i}|\mathbf {X}_{i})}$, $\overline{U}_{n}=n^{-1} \sum _{i=1}^{n} U_{i}$, and $\overline{W}_{n}=n^{-1} \sum _{i=1}^{n} W_{i}$. Through proofs and simulations, Jiang et al. [7] showed consistency and asymptotic normality of the proposed jackknife estimator of the value function. Note that we are ignoring the contribution to the limiting distribution due to estimating the propensity scores. It appears from our simulation study that this omission does not harm the performance of our method for a range of sample sizes. Evaluating this question theoretically is beyond the scope of this paper.

In the presence of dropouts or administrative censoring, the unobserved events bring some complexity to statistical analysis. Specifically, the presence of informative censoring (i.e., the censoring time is dependent on the failure time) may incur bias in both survival times and censoring probabilities. Thus, the higher the proportion of censoring in the data, the more caution is to be taken during the estimations procedures. One approach that is commonly used to address the issues arising from censoring is the inverse probability of censoring weighted estimator (IPCW). The IPCW was initially developed to address bias in survival probabilities due to censoring [21–24]. The IPCW estimator corrects for bias by giving zero weight to censored observations and extra weights to uncensored observations. The weights are assigned to observations with similar characteristics to the censored observations and are usually estimated by logistic regression or Cox proportional hazards regression. Applying the IPCW recreates a potential scenario of no censoring and the re-weighted sample obtained is asymptotically unbiased. However, IPCW does not address possible models misspecification that may remain when parametric or semi-parametric estimation methods are employed.

### Jackknife estimator of the value for right-censored data

#### Definition of the jackknife estimator of the value function for right-censored data

We propose an extension of the jackknife value estimator developed by Jiang et al [7] in the presence of right censoring. Our jackknife value estimator corrects for the censoring-induced bias by applying the IPCW method to the estimator expressed in (1). The proposed jackknife value estimator has the following form:

2 $$\begin{aligned} \widehat{V}^{jk} (\hat{d}_{n}) = \frac{ \sum _{i=1}^{n} T_{i} \frac{1\left\{A_{i}=\hat{d}^{(-i)}_{n} (\mathbf {X}_{i})\right\}}{{P}(A_{i}|\mathbf {X}_{i})} \frac{\delta _{i}}{{S}_{c}(T_i|\mathbf {X}_{i})} }{ \sum _{i=1}^{n} \frac{1\left\{A_{i}=\hat{d}^{(-i)}_{n} (\mathbf {X}_{i})\right\}}{{P}(A_{i}|\mathbf {X}_{i})} \frac{\delta _{i}}{{S}_{c}(T_i|\mathbf {X}_{i})} }, \end{aligned}$$

where $\hat{d}^{(-i)}_{n}$ represents the treatment rule estimated from a training set of size *n* with the *i*-th observation left out, ${P}(A_{i}|\mathbf {X}_{i})$ is the propensity score of the test set *i*, ${S}_{c}(t|\mathbf {X}_{i})$ the probability that the *i*-th observation has not been censored by time *t* conditional on $\mathbf {X}_{i}$, and $\delta _i=I(T_i \le C_i)$. Here, we assume that ${P}(A_{i}|\mathbf {X}_{i})$ and ${S}_{c}(t|\mathbf {X}_{i})$ are known. However, in practice, ${P}(A_{i}|\mathbf {X}_{i})$ and ${S}_{c}(t|\mathbf {X}_{i})$ are sometimes unknown and are estimated by regression models. Once obtained, the estimated propensity scores and censoring probabilities are plugged in the expression for the jackknife estimator in (2).

The estimated variance of the jackknife value estimator is

$$\begin{aligned} \widehat{Var}\left[\widehat{V}^{jk} (\hat{d}_{n})\right] = \frac{1}{n (n-1)} \sum _{i=1}^{n} \left(R^{jk}_{i}\right)^2, \end{aligned}$$

where $R_i^{jk}= \frac{1}{\overline{W}_{n}} U_i - \frac{\overline{U}_{n}}{\overline{W}^2_{n}} W_i$ is the bias-corrected form of value function inspired by the influence function, with $U_i= T_{i} \frac{1\left\{A_{i}=\hat{d}^{(-i)}_{n} (\mathbf {X}_{i})\right\}}{{P}(A_{i}|\mathbf {X}_{i})} \frac{\delta _{i}}{{S}_{c}(T_i|\mathbf {X}_{i})}$, $W_i= \frac{1\left\{A_{i}=\hat{d}^{(-i)}_{n} (\mathbf {X}_{i})\right\}}{{P}(A_{i}|\mathbf {X}_{i})} \frac{\delta _{i}}{{S}_{c}(T_i|\mathbf {X}_{i})}$, $\overline{U}_{n}=n^{-1} \sum _{i=1}^{n} U_{i}$, and $\overline{W}_{n}=n^{-1} \sum _{i=1}^{n} W_{i}$.

We note that, as before, we are ignoring the potential variability due to estimating ${P}(A_{i}|\mathbf {X}_{i})$ and ${S}_{c}(t|\mathbf {X}_{i})$, but our simulations verify that the approach works acceptably well for a range of sample sizes considered.

#### Estimation of the optimal treatment rule

The optimal treatment rule $d_n^{opt}$ maps the patient-level covariates $\mathcal {X}$ to the binary treatment space $\{ 0, 1 \}$. $d_n^{opt}$ is selected among a class of all possible treatment rules $\mathcal {D}$ that maximize the value of a potential outcome when applied to future patients [2].

A wide range of machine learning approaches has been used to obtain $\hat{d}_{n}^{opt}$, the estimated optimal treatment rule when the outcome is right-censored. Goldberg and Kosorok [15] proposed a Q-learning algorithm with backward recursion for the multi-stage setting. Zhao et al. [11] developed a doubly robust outcome-weighted learning approach that uses IPCW and requires the correct specification of either the survival or the censoring model. Cui et al. [12] further proposed an extension of outcome weighted learning which relaxes the previous requirements by considering a tree-based approach. In this manuscript, we opt for using random survival forests [25] (RSF) and the Cox proportional hazards model (COX) as our estimation approaches. Random survival forests is one of the most commonly used tree-based methods for survival analysis. Tree-based methods are widely used because they are flexible methods that do not make distributional assumptions for the failure times. Even though Cox proportional hazards models [26] make some assumptions on the distribution of the failure times, they are robust to misspecification and remain the most commonly used regression method for survival analysis [27].

Using each of the selected estimation approaches, we obtain the estimated restricted mean survival times (RMST) [28] corresponding to each treatment option. The optimal treatment for a patient *i* is then obtained by the treatment that yields the larger area under the curve.

Under mild assumptions, we prove that the proposed jackknife estimator for right-censored data is consistent.

### Consistency of the jackknife estimator for right-censored data

In this section, we show proof of the consistency of the jackknife estimator. First, we make the following two assumptions.

#### Assumption 1

$$E[P_{X}(\hat{d}_{n} (\mathbf {X}) \ne \hat{d}_{n-1} (\mathbf {X}) )] \longrightarrow 0$$

as $$n\rightarrow \infty$$

#### Assumption 2

$$E\Big [\frac{\mathbf {T}^{2} + 1}{P^2(A|\mathbf {X}) \; S^2_{c} (T|\mathbf {X}) } \Big ] < \infty$$

Assumption 1 makes the assumption that the treatment rules based on samples of size *n* and $n-1$ are asymptotically equal in probability. Assumption 2 assumes that the expectation of the second moment of the outcome, adjusted by the propensity score and the probability of censoring, is finite.

#### Theorem 1

Given the above assumptions,

3 $$\begin{aligned}&\frac{\sum _{i=1}^{n} T_{i} \frac{1\left\{A_{i}=\hat{d}^{(-i)}_{n} (\mathbf {X}_{i})\right\}}{P(A_{i}|\mathbf {X}_{i})} \frac{\delta _{i}}{S_{c}(T_{i}|\mathbf {X}_{i})} }{ \sum _{i=1}^{n} \frac{1\left\{A_{i}=\hat{d}^{(-i)}_{n} (\mathbf {X}_{i})\right\}}{P(A_{i}|\mathbf {X}_{i})} \frac{\delta _{i}}{S_{c}(T_{i}|\mathbf {X}_{i})} } - E\big [ T|A= \hat{d}_{n} (\mathbf {X}) \big ]\nonumber \\&\qquad \qquad \qquad \qquad \qquad \qquad \qquad \qquad \overset{p}{\longrightarrow } 0 \; \text {as} \; n \rightarrow \infty , \end{aligned}$$

when $P(A|\mathbf {X})$ and $S_{c}(T|\mathbf {X})$ are known.

The complete proof is shown in Additional file 1.

### Z-test for comparing two ITRs in the presence of right-censored data

We build a test to compare optimal treatment rules from two estimation approaches. Similarly to Jiang et al. [7], the test statistic is expressed as:

4 $$\begin{aligned} T(\hat{d}_{n,1}, \hat{d}_{n,2}) = \frac{\widehat{V}(\hat{d}_{n,1}) -\widehat{V}(\hat{d}_{n,2}) }{\sqrt{\frac{\sum _{i=1}^{n} (R_{\text {1,i}}-R_{\text {2,i}})^2}{n(n-1)}}} , \end{aligned}$$

where $\widehat{V}(\hat{d}_{n,1})$ and $\widehat{V}(\hat{d}_{n,2})$ are the estimated value functions for the estimated optimal treatment rules obtained from the two estimation approaches. $R_{\text {1,i}}$ and $R_{\text {2,i}}$ are the bias-corrected, influence function-inspired value function of the *i*-th individual under these treatment rules.

The *p*-value for the test is obtained as $p= 2 \; \int _{\left| T \right| }^{\infty } f(z) \; dz$, where *f* is the density of the standard normal distribution. We define the power of the test as the estimated proportion of the total number of simulations whose *p*-values are under 0.05.

Through simulations, we study the asymptotic properties of the jackknife estimator. We provide Q-Q plots for the distribution of the test statistic above and boxplots for the jackknife value estimate obtained after 500 replications.

## Simulation studies

### Simulation settings

We carry out simulations to assess the performance of the proposed jackknife estimator of the value function. The binary treatment $A \in \{0, 1 \}$ is assigned with equal probabilities. We generate five covariates $X_1, \ldots, X_5$ independently from a uniform (*U*[0, 1]) distribution. We obtain and compare the estimated optimal treatment rules with random survival forests (RSF) and proportional hazards regression (COX). We also compare the RSF to the zero-order model (ZOM) or one-size-fits-all, which assigns the best single treatment to all patients. For each estimation approach, using the five generated covariates, we estimate the optimal treatment as detailed in the Methods section. The IPCWs are obtained by Cox proportional hazards models using all five covariates and their interactions with the binary treatment. To control for potential extreme outliers in the estimated values, we apply a $90\%$ winsorization — trimming of the top and bottom $5\%$ — to the estimated censoring probabilities [29]. The propensity scores are obtained with logistic regression where we regress the binary treatment on all five covariates. Although they are known a priori in our simulations, we use the estimated propensity scores to correctly reflect variability in the estimation of the value function. We train the RSF with the R package *randomforestSRC* [30] with the default tuning parameters. We use the *survival* [31] package to conduct the proportional hazards regressions.

We consider three simulation scenarios where the true survival ( $\tilde{T}$) and censoring times (*C*) are conditionally independent. We use log-transformed survival and censoring times in all our analyses. For each scenario, we perform the simulations 500 times for sample sizes of 200, 400 and 800, and consider the average censoring rates of $0 \%$, $10\%$, $20\%$, $40\%$. For all models, the errors for the failure times ($\epsilon$) are generated from a normal distribution with mean 0 and standard deviation 0.2, and the errors for the censoring times ($\xi$) are generated from a normal distribution with mean 0 and standard deviation 0.5. For the proportional hazards model, the baseline hazard function $\lambda _0 {(t)} = 2t$.

The simulation scenarios are detailed below.

$${\textbf {Scenario 1.}}$$
$\tilde{T}$ is generated from the accelerated failure time model. $\tau$ is 1.8 and $\alpha$ is 0.5, 0.22 and $-0.14$ for $10\%$, $20\%$ and $40\%$ average censoring rates respectively: $\log (\tilde{T})= -0.2 -0.5X_{1} +0.5X_{2} + 0.4X_{3} + (0.3 -0.1X_{1} -0.6X_{2} + 0.1X_{3})A + \epsilon$, $\log (C)= \alpha -0.1X_1 +0.2X_2 +0.2X_3 + (0.5 -0.1X_1 -0.6X_2 + 0.3X_3)A + \xi$. The value of the optimal ITR is approximately 1.105.
$${\textbf {Scenario 2.}}$$
$\tilde{T}$ is generated from the accelerated failure time model with tree-structured effects. $\tau$ is 8 and $\alpha$ is 0.5, $-0.25$ and $-1.18$ for $10\%$, $20\%$ and $40\%$ average censoring rates respectively: $\log (\tilde{T})= X_1 + I(X_2> 0.5) I(X_3 > 0.5) + (0.3 - X_1)A + 2\{I(X_4< 0.3) I(X_5 < 0.3) \}A + \epsilon$, $\log (C)= \alpha -X_1 +2X_2 +2X_3 + (5 -X_1 -6X_2 +3X_3)A + \xi$. The value of the optimal ITR is approximately 3.590.
$${\textbf {Scenario 3.}}$$
$\tilde{T}$ is generated from the Cox proportional hazards model. $\tau$ is 2.5 and $\alpha$ is $-0.05$, $-0.40$ and $-0.93$ for $10\%$, $20\%$ and $40\%$ average censoring rates respectively: $\lambda _{\tilde{T}}{(t \; | \; A,X)} = \lambda _0 {(t)} \; \text {exp}\{-0.2 + 0.75 X_1^{1.5} - 0.25 X_2 + (1.6 - 1.4 X_1^{0.5} - 2.4 X_2^2) A \}$, $\log (C)= \alpha +0.5X_1 +X_2 +0.3X_3 + 0.1X_4 + (0.1 +0.5X_1 -X_2 +0.3X_3)A + \xi$. The value of the optimal ITR is approximately 1.144.

One inconvenience of using the jackknife estimator is that it becomes rapidly computationally intensive as the sample size increases. We attempt to mitigate this issue for large sample sizes by performing a partial jackknife (LOOCV) on a random subset $r, r \le n$ of the original sample. Thus, we compute the jackknife value estimator in (2) for $n=200$ and $n=400$ and opted for the partial jackknife for $n=800$—for each replicate, we apply the expression of the jackknife value estimator on a randomly selected subset $r=500$ of the initial sample $n=800$. Through our work with simulated data sets, we observe that using the partial jackknife requires up to $40 \%$ less computation time than using the “full jackknife” for a sample of size $n=800$.

### Results

**Fig. 1:**
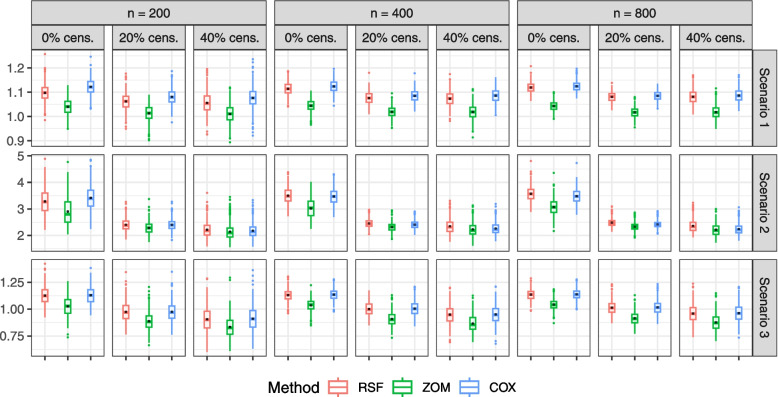
Boxplots of the estimated values obtained across 500 simulations. “cens.” stands for censoring. The red, green and blue plots represent the COX, RSF and ZOM respectively. Only are shown plots for 0%, 20 % and 40% censoring

Table 1 shows the power of the test statistic expressed in (4). We provide a table with the value of the optimal ITR and the estimated values for each scenario under the $0 \%$ censoring case in Additional file 2. We compare the estimated optimal individualized treatment rules obtained from RSF and COX on the one hand, and RSF and ZOM on the other hand. RSF outperforms ZOM in all scenarios. RSF tends to show an increased performance over COX as the event times stem from more non-linear distributions. Figure 1 illustrates the boxplots of the estimated values obtained using each estimation method for all three scenarios.

**Table 1:** Estimated power of the jackknife estimator based on 500 simulations. RSFvCOX and RSFvZOM correspond to the comparison between RSF and COX, and between RSF and ZOM respectively

		n = 200		n = 400		n = 800	
Scenario	Censoring	RSFvCOX	RSFvZOM	RSFvCOX	RSFvZOM	RSFvCOX	RSFvZOM
1	0 %	0.164	0.452	0.114	0.878	0.066	0.968
	$\approx 10$ %	0.124	0.414	0.092	0.786	0.066	0.914
	$\approx 20$ %	0.122	0.332	0.080	0.682	0.050	0.860
	$\approx 40$ %	0.106	0.234	0.090	0.474	0.054	0.680
2	0 %	0.094	0.362	0.074	0.928	0.230	0.992
	$\approx 10$ %	0.074	0.202	0.070	0.458	0.170	0.562
	$\approx 20$ %	0.040	0.128	0.096	0.234	0.172	0.348
	$\approx 40$ %	0.076	0.066	0.124	0.104	0.212	0.206
3	0 %	0.046	0.442	0.054	0.732	0.060	0.830
	$\approx 10$ %	0.034	0.372	0.064	0.642	0.060	0.764
	$\approx 20$ %	0.048	0.294	0.062	0.540	0.052	0.590
	$\approx 40$ %	0.034	0.126	0.046	0.242	0.046	0.320

In scenario 1 and across all censoring mechanisms, COX performs better than RSF for $n=200$. As the sample size increases, the two estimation approaches show similar performances. As expected, RSF always performs better than ZOM, with enhancing performance as the sample size increases and a decreasing performance as the censoring proportion gets larger.

In scenario 2, as the failure times are generated with a tree-based structure, COX performs slightly better than RSF for $n=200$. For $n=400$ both methods perform similarly and RSF outperforms COX for $n=800$. We observe a similar pattern as in scenario 1 when we compare RSF to ZOM. RSF does better than ZOM as the sample size increases and the performance decreases as more censoring is introduced in the data.

When the data are generated from a proportional hazards model in scenario 3, we observe similar results to scenario 1. COX consistently performs better than RSF for all censoring mechanisms and across sample sizes, while RSF always does better than ZOM.

We investigated the size of the proposed Z-test under the null hypothesis. The null hypothesis is that the value (or mean outcome) is the same for the two estimation methods being compared. Across all three scenarios, we modified the posited failure time models to make one treatment option clearly more favorable than the other treatment option. Under these settings, we assess whether the type I error is preserved for the proposed Z-test. *log*(*C*) and $\tau$ remain the same as in the previously presented simulation scenarios, and we tuned $\alpha$ to achieve the desired proportion of censored observation in each simulation. We present the slightly modified scenarios in Additional file 3. In table 2, we present the *p*-values of the tests, along with the Monte Carlo errors derived from these models. It is important to note that, for scenario 2 and 40% censoring, we do not achieve the desired test size of around 0.05. We suspect that, for high censoring rates, and when the failure time model is not linear, a sample size larger than the ones considered in our simulations is necessary to achieve a type I error.

**Table 2:** Estimated test size of the jackknife estimator based on 500 simulations. Size of the test & Monte Carlo Error ( $\times 10^3$). RSFvCOX and RSFvZOM correspond to the comparison between RSF and COX, and between RSF and ZOM respectively

		n = 200		n = 400		n = 800	
Scenario	Censoring	RSFvCOX	RSFvZOM	RSFvCOX	RSFvZOM	RSFvCOX	RSFvZOM
1	0 %	0.052 (9.9)	0.064 (11.0)	0.044 (9.2)	0.048 (9.6)	0.020 (6.3)	0.020 (6.3)
	$\approx 10$ %	0.056 (10.3)	0.090 (12.8)	0.088 (12.7)	0.110 (14.0)	0.050 (9.7)	0.050 (9.7)
	$\approx 20$ %	0.046 (9.4)	0.070 (11.4)	0.080 (12.1)	0.100 (13.4)	0.062 (10.8)	0.064 (10.9)
	$\approx 40$ %	0.052 (9.9)	0.074 (11.7)	0.064 (10.9)	0.110 (14.0)	0.060 (10.6)	0.066 (11.1)
2	0 %	0.032 (7.9)	0.044 (9.2)	0.034 (8.1)	0.068 (11.2)	0.04 (8.8)	0.038 (8.5)
	$\approx 10$ %	0.080 (12.1)	0.070 (11.4)	0.066 (11.1)	0.088 (12.7)	0.052 (9.9)	0.044 (9.2)
	$\approx 20$ %	0.080 (12.1)	0.088 (12.7)	0.076 (11.8)	0.098 (13.3)	0.076 (11.8)	0.110 (14.0)
	$\approx 40$ %	0.116 (14.3)	0.100 (13.4)	0.154 (16.1)	0.148 (15.9)	0.170 (16.8)	0.170 (16.8)
3	0 %	0.040 (8.8)	0.066 (11.1)	0.062 (10.8)	0.118 (14.4)	0.088 (12.7)	0.098 (13.3)
	$\approx 10$ %	0.052 (9.9)	0.044 (9.2)	0.054 (10.1)	0.100 (13.4)	0.082 (12.3)	0.082 (12.3)
	$\approx 20$ %	0.034 (8.1)	0.036 (8.3)	0.042 (9.0)	0.092 (12.9)	0.048 (9.6)	0.048 (9.6)
	$\approx 40$ %	0.040 (8.8)	0.044 (9.2)	0.028 (7.4)	0.070 (11.4)	0.050 (9.7)	0.060 (10.6)

**Fig. 2:**
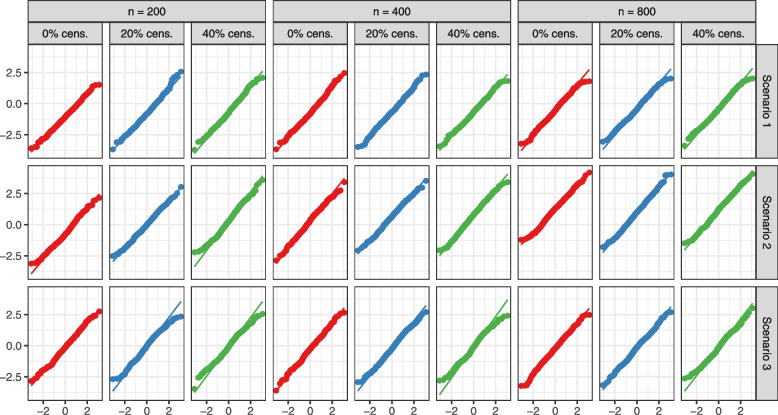
Q-Q plots of the distribution of the jackknife test statistic for comparing RSF to COX across 500 simulations versus the standard normal distribution. “cens.” stands for censoring. Only are shown plots for 0%, 20 % and 40% censoring

**Fig. 3:**
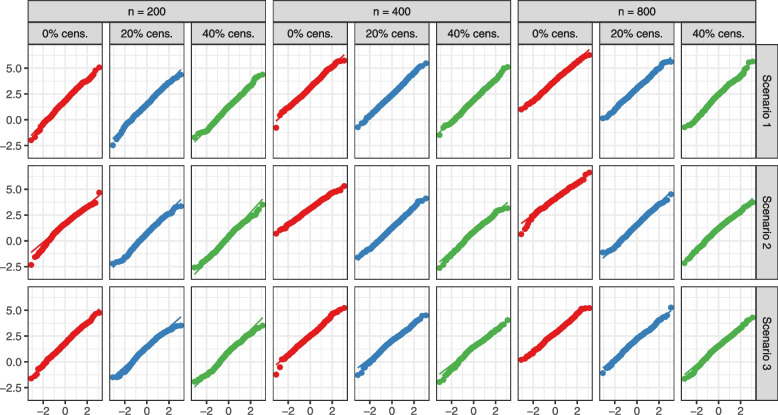
Q-Q plots of the distribution of the jackknife test statistic for comparing RSF to ZOM across 500 simulations versus the standard normal distribution. “cens.” stands for censoring. Only are shown plots for 0%, 20 % and 40% censoring

We studied the normality of the jackknife estimators under each scenario and censoring mechanism. Figures 2 and 3 contain the Q-Q plots for the jackknife test statistic expressed in equation (4) with $90\%$ winsorized censoring probabilities. We show the plots for $0 \%$, $20\%$ and $40\%$ censoring. When the proportion of censoring is relatively small ($\approx 10\%$), we observe some results analogous to the no censoring case. Across all scenarios, and when there is no or minimal censoring, we observe that the test statistic has approximately a standard normal distribution. Even as the censoring proportion increases and the failure times are of more complex structures, the test statistic remains normally distributed. Trimming the top and bottom $5\%$ weights permits avoiding extreme outliers that would cause deviations from normality. In general, departures from normality in the tails are not uncommon in survival analysis when censoring is high.

## Data application

We illustrate our proposed method using non-small-cell lung cancer data [32]. The Phase III randomized trial was conducted to investigate the duration of therapy that would maximize survival. Patients with advanced non-small-cell lung cancer were recruited and randomized to either four cycles of carboplatin/paclitaxel or continuous therapy with carboplatin/paclitaxel until disease progression. The trial enrolled 230 participants; however, our analysis data set contains information for 224 participants with complete data. In this sample, 115 participants were assigned to continuous therapy with carboplatin/paclitaxel until disease progression and 109 participants were assigned to four cycles of carboplatin/paclitaxel. We consider five covariates in our data analysis: performance status, cancer stage, race, sex, and age. The censoring rate was around 32%.

We apply our proposed method to the analytic sample. We set $\tau$ = 500 days, thus truncating the six highest survival times to 500 days. Similar to the simulations settings, the IPCWs are estimated by Cox proportional hazards models using all five covariates and their interactions with the binary treatment. The propensity scores are obtained via logistic regressions where we regress the binary treatment on all five covariates. We trained the RSF with default tuning parameters. With each RSF, COX, and ZOM, we compared RMST for both treatment groups to obtain the optimal ITRs. We then computed the corresponding jackknife value estimates, along with corresponding standard errors. We also computed the Z-test comparing RSF to COX, RSF to ZOM and COX to ZOM.

As illustrated by their value estimates (Table 3), RSF performed the worst, followed by COX, and ZOM performed the best. RSF performed worse than either COX ($p=0.06$) or ZOM ($p=0.05$), and ZOM performed better than COX ($p=0.46$).

**Table 3:** Jackknife value estimates and standard errors for the non-small cell lung cancer analytic sample

	RSF	COX	ZOM
Value	227.90	244.15	252.33
SE	18.61	19.38	18.21

There are a few possible explanations for why ZOM provided the best performance. First, we may not have a sufficiently diverse pool of patients in our analytic sample; indeed, we know that precision medicine requires heterogeneity to perform well. It appears that tailoring is not needed in this case, and all patients are recommended to receive “continuous therapy with carboplatin/paclitaxel until disease progression”. Second, the sample size was not very large, and we only used five tailoring variables in our analysis. Having more features available to proceed with a thorough variable selection would have benefited RSF, as random forests are known to be highly flexible. ZOM selects “continuous therapy with carboplatin/paclitaxel until disease progression” as the best single treatment, which is consistent with the findings of the paper from [32] discussing the results from the trial.

## Discussion

In this article, we proposed an extension of the jackknife method to estimate value functions and optimal treatment rules when outcomes are right-censored survival data. The method shows strong performance for small to mild censoring rates. When the outcome has a high proportion of censoring, trimming the top and bottom $5\%$ of the estimated censoring probabilities leads to satisfactory results.

In our method, we used the complete data to estimate the inverse probability of censoring and propensity weights before applying the jackknife method. Unfortunately, it was not possible to estimate these weights with test sets of only one observation within the jackknife procedure. We would have had an empty set each time the left-out observation was censored, and thus, would have been unable to compute the weights. Alternatively, we could have estimated the weights under settings of no censoring. However, this naive approach also has obvious limitations. While we expect this approach in estimating the censoring probabilities to have some effects on our estimation results, it is not clear how significant these effects are based on our simulations.

Future research should investigate the use of leave-five-out- or leave-ten-out-cross-validation (instead of the LOOCV) to increase the predictive performance in a high censoring setting. Also, it would be of interest to determine a systematic procedure for defining the truncation point needed for the RMST to improve the method performance in the presence of high censoring. Finally, a theoretical understanding of the reasons why the potential variability created by estimating the propensity scores and the censoring probabilities does not affect the performance of our estimator, remains an open question.

## Supplementary information

**Additional file 1:** Proof for the consistency of the proposed jackknife estimator for right-censored data.

**Additional file 2:** Values obtained based on non censored data according to the true and estimated treatment rules.

**Additional file 3:** Simulations scenarios for assessing the Type I error.

## Data Availability

The data set analyzed, and the R codes for the simulations performed in the present manuscript are available from the corresponding author on reasonable request.

## Abbreviations

COX: Cox proportional hazards model
CV: Cross-validation
LOOCV: Leave-one-out-cross-validation
IPCW: Inverse probability of censoring weighting
ITR: Individualized treatment rule
RMST: Restricted mean survival time
RSF: Random survival forest
ZOM: Zero-order model
