# Supplementary material for: Model selection for survival individualized treatment rules using the jackknife estimator
Source: BMC Med Res Methodol. 2022 Dec 22;22:328. doi: 10.1186/s12874-022-01811-6 (PMC9773469; doi:10.1186/s12874-022-01811-6)
Supplement: Supplementary file 1 — Additional file 1: Proof for the consistency of the proposed jackknife estimator for right-censored data. [file 12874_2022_1811_MOESM1_ESM.pdf]

# SUPPLEMENTARY MATERIALS

# Model Selection for Individualized Treatment Rules Using the Jackknife Estimator

Gilson D Honvoh[1,2], Hunyong Cho[1], and Michael R. Kosorok[1]

[1]Department of Biostatistics, University of North Carolina at Chapel Hill
[2]Department of Physical Medicine & Rehabilitation, School of Medicine, University of North Carolina at Chapel Hill

## Additional file 1: Consistency of the jackknife estimator for right-censored data

We show proof of the consistency of the jackknife estimator. First, we make the following two assumptions

$$E[P_X(\hat{d}_n(\mathbf{X}) \neq \hat{d}_{n-1}(\mathbf{X}))] \longrightarrow 0 \quad (1)$$

$$E\Big[\frac{\mathbf{T}^2 + 1}{P^2(A|\mathbf{X})\; S_c^2(T|\mathbf{X})}\Big] < \infty. \quad (2)$$

(1) makes the assumption that the treatment rules based on samples of size $n$ and $n-1$ are asymptotically equal in probability. (2) assumes that the expectation of the second moment of the outcome, adjusted by the propensity score and the probability of censoring, is finite.

Given the above assumptions, we make the proposition that

$$\frac{\sum_{i=1}^n T_i \frac{1\{A_i=\hat{d}_n^{(-i)}(\mathbf{X}_i)\}}{P(A_i|\mathbf{X}_i)} \frac{\delta_i}{S_c(T_i|\mathbf{X}_i)}}{\sum_{i=1}^n \frac{1\{A_i=\hat{d}_n^{(-i)}(\mathbf{X}_i)\}}{P(A_i|\mathbf{X}_i)} \frac{\delta_i}{S_c(T_i|\mathbf{X}_i)}} - E\big[T|A=\hat{d}_n(\mathbf{X})\big] \xrightarrow{p} 0, \text{ when } P(A|\mathbf{X}) \text{ and} S_c(T|\mathbf{X}) \text{ are known.}$$

Let $U_i = T_i \frac{1\{A_i = \hat{d}_n^{(-i)}(\mathbf{X}_i)\}\delta_i}{P(A_i|\mathbf{X}_i)\ S_c(T|\mathbf{X}_i)}$, $W_i = \frac{1\{A_i = \hat{d}_n^{(-i)}(\mathbf{X}_i)\}\delta_i}{P(A_i|\mathbf{X}_i)\ S_c(T|\mathbf{X}_i)}$, $U_n = n^{-1}\sum_{i=1}^n U_i$, $W_n = n^{-1}\sum_{i=1}^n W_i$, and

$$\mu_n = E[U_n] = n^{-1}\sum_{i=1}^n E\Big[T_i \frac{1\{A_i = \hat{d}_n^{(-i)}(\mathbf{X}_i)\}\delta_i}{P(A_i|\mathbf{X}_i)\ S_c(T_i|\mathbf{X}_i)}\Big] = E\Big[T \frac{1\{A = \hat{d}_{n-1}(\mathbf{X})\}}{P(A|\mathbf{X})\ S_c(T|\mathbf{X})}\Big].$$

We note $\tilde{\mu}_n = E\Big[T \frac{1\{A = \hat{d}_n(\mathbf{X})\}}{P(A|\mathbf{X})\ S_c(T|\mathbf{X})}\Big]$, and then

$$\begin{aligned}
\mu_n - \tilde{\mu}_n &= E\Big[\frac{T}{P(A|\mathbf{X})\ S_c(T|\mathbf{X})}\big(1\{A = (\hat{d}_{n-1}\mathbf{X})\} - 1\{A = \hat{d}_n(\mathbf{X})\}\big)\Big] \\
&\le M\ E\Big[\big(1\{A = \hat{d}_{n-1}(\mathbf{X})\} - 1\{A = \hat{d}_n(\mathbf{X})\}\big)\Big] \\
&\quad + E\Big[\frac{T}{P(A|\mathbf{X})\ S_c(T|\mathbf{X})}\ 1\Big\{\frac{T}{P(A|\mathbf{X})\ S_c(T|\mathbf{X})} > M\Big\}\Big] \longrightarrow 0
\end{aligned}$$

where $M < \infty$ is a constant. The convergence of the first term is based on assumption (1) and the convergence of the second term is based on assumption (2).

We also have that

$$
\begin{aligned}
Var[U_n] &= n^{-1} Var\Big\{\sum_{i=1}^{n} U_i\Big\} \\
&= n^{-2}\sum_{i=1}^{n}\sum_{j=1}^{n}\big[E\big(U_iU_j\big) - E(U_i)E(U_j)\big)\big] \\
&= n^{-2}\sum_{i=1}^{n}\sum_{j=1}^{n}\Big[E\Big(T_iT_j\delta_i\delta_j\frac{1\{A_i=\hat{d}_n^{(-i)}(\mathbf{X}_i)\}1\{A_j=\hat{d}_n^{(-j)}(\mathbf{X}_j)\}}{P(A_i|\mathbf{X}_i)\ S_c(T_i|\mathbf{X}_i)P(A_j|\mathbf{X}_j)\ S_c(T_j|\mathbf{X}_j)}\Big) - \mu_n^2\Big] \\
&= n^{-2}\sum_{i=1}^{n}\sum_{j=1}^{n}\Big[E\Big(T_iT_j\delta_i\delta_j\frac{1\{A_i=\hat{d}_n^{(-i,-j)}(\mathbf{X}_i)\}1\{A_j=\hat{d}_n^{(-i,-j)}(\mathbf{X}_j)\}}{P(A_i|\mathbf{X}_i)\ S_c(T_i|\mathbf{X}_i)P(A_j|\mathbf{X}_j)\ S_c(T_j|\mathbf{X}_j)}\Big)\Big] \\
&\quad -\mu_n^2 + o_p(1) \\
&= n^{-2}\sum_{i}\Big[E\Big\{\Big(\frac{T_i\delta_i}{P(A_i|\mathbf{X}_i)\ \ S_c(T_i|\mathbf{X}_i)}\Big)^2 1\{A_i=\hat{d}_n^{(-i)}(\mathbf{X}_i)\}\Big\}\Big] + \\
&\quad n^{-2}\sum_{i=1}^{n}\sum_{j\neq i}E\Big[E\Big(T_iT_j\delta_i\delta_j\frac{1\{A_i=\hat{d}_n^{(-i,-j)}(\mathbf{X}_i)\}1\{A_j=\hat{d}_n^{(-i,-j)}(\mathbf{X}_j)\}}{P(A_i|\mathbf{X}_i)\ S_c(T_i|\mathbf{X}_i)P(A_j|\mathbf{X}_j)\ S_c(T_j|\mathbf{X}_j)}\Big|\mathbb{D}^{-i,-j}\Big)\Big] \\
&\quad -\mu_n^2 + o_p(1) \\
&= n^{-2}\sum_{i}\Big[E\Big\{\Big(\frac{T_i\delta_i}{P(A_i|\mathbf{X}_i)\ \ S_c(T_i|\mathbf{X}_i)}\Big)^2 1\{A_i=\hat{d}_n^{(-i)}(\mathbf{X}_i)\}\Big\}\Big] + \\
&\quad n^{-2}\sum_{i=1}^{n}\sum_{j\neq i}E\Big[E\Big(T_i\delta_i\frac{1\{A_i=\hat{d}_n^{(-i,-j)}(\mathbf{X}_i)\}}{P(A_i|\mathbf{X}_i)\ S_c(T_i|\mathbf{X}_i)}\Big|\mathbb{D}_n^{-i,-j}\Big) \\
&\qquad\qquad \times E\Big(T_j\delta_j\frac{1\{A_j=\hat{d}_n^{(-i,-j)}(\mathbf{X}_j)\}}{P(A_j|\mathbf{X}_j)\ S_c(T_j|\mathbf{X}_j)}\Big|\mathbb{D}_n^{-i,-j}\Big)\Big] - \mu_n^2 + o_p(1) \\
&= n^{-2}\sum_{i}\Big[E\Big\{\Big(\frac{T_i\delta_i}{P(A_i|\mathbf{X}_i)\ \ S_c(T_i|\mathbf{X}_i)}\Big)^2 1\{A_i=\hat{d}_{n-1}(\mathbf{X}_i)\}\Big\}\Big] + \\
&\quad n^{-2}\sum_{i=1}^{n}\sum_{j\neq i}E\Big[E\Big(T_i\delta_i\frac{1\{A_i=\hat{d}_{n-2}(\mathbf{X}_i)\}}{P(A_i|\mathbf{X}_i)\ S_c(T_i|\mathbf{X}_i)}\Big|\hat{d}_{n-2}\Big)\Big]^2 - \mu_n^2 + o_p(1) \\
&= n^{-2}\left\{cn + n(n-1)E\Big[E\Big(T_1\frac{1\{A_1=\hat{d}_{n-2}(\mathbf{X}_1)\}}{P(A_1|\mathbf{X}_1)\ S_c(T_1|\mathbf{X}_1)}\Big|\hat{d}_{n-2}\Big)\Big]^2\right\} - \mu_n^2 + o_p(1) \\
&= o_p(1),
\end{aligned}
$$

where $\mathbb{D}_n^{-i,-j}$ is the data set of size $n$ with the $i$th and $j$th elements excluded, and $c < \infty$ is some constant by the second assumption (2).

Thus, we have shown that

$$\begin{aligned} E[U_n - \tilde{\mu}_n] &\longrightarrow 0, \text{and} \\ Var[U_n] &\longrightarrow 0. \end{aligned}$$

Similarly, we can show that

$$\begin{aligned} E[W_n - 1] &\longrightarrow 0, \text{and} \\ Var[W_n] &\longrightarrow 0. \end{aligned}$$

By the WLLN, $U_n - \tilde{\mu}_n \xrightarrow{p} 0$ and $W_n - 1 \xrightarrow{p} 0$, which implies that

$$\frac{U_n}{W_n} - \tilde{\mu}_n \xrightarrow{p} 0.$$

This completes the proof since $\tilde{\mu}_n = E\Big[T \frac{1\{A = \hat{d}_n(\mathbf{X})\}}{P(A|\mathbf{X})\, S_c(T|\mathbf{X})}\Big] = E\big[T|A = \hat{d}_n(\mathbf{X})\big]$.
