# Supplementary material for: Model selection for survival individualized treatment rules using the jackknife estimator
Source: BMC Med Res Methodol. 2022 Dec 22;22:328. doi: 10.1186/s12874-022-01811-6 (PMC9773469; doi:10.1186/s12874-022-01811-6)
Supplement: Supplementary file 2 — Additional file 2: Values obtained based on non censored data according to the true and estimated treatment rules. [file 12874_2022_1811_MOESM2_ESM.pdf]

# Additional file 2: Values obtained based on non censored data according to the true and estimated treatment rules

For each scenario described in our simulation settings, we generated 500 data sets of size $n = 100,000$. For each data set, we obtained the value of the optimal treatment rule and averaged them across the simulated data sets. The values of the optimal ITR and the ITRs estimated with each method are shown in the table below.

**Table**: Values of the optimal ITR and the ITRs estimated with RSF, COX and ZOM. "Opt." is the optimal ITR obtained as described above. For RSF, COX and ZOM, we calculated the averages of the estimated values over the data sets described in our simulation settings.

| | | n = 200 | | | n = 400 | | | n = 800 | | |
|---|---|---|---|---|---|---|---|---|---|---|
| Scenario | Opt. | RSF | COX | ZOM | RSF | COX | ZOM | RSF | COX | ZOM |
| 1 | 1.105 | 1.097 | 1.122 | 1.040 | 1.114 | 1.123 | 1.044 | 1.119 | 1.124 | 1.053 |
| 2 | 3.590 | 3.287 | 3.419 | 2.904 | 3.503 | 3.475 | 3.022 | 3.576 | 3.483 | 3.070 |
| 3 | 1.144 | 1.127 | 1.130 | 1.026 | 1.131 | 1.136 | 1.035 | 1.136 | 1.139 | 1.043 |

As expected, the values increase as the samples get larger. We observe that COX provides the highest values for scenarios 1 and 3, while RSF performs well in scenario 2. These observations are all in agreement with the results previously obtained from our simulations and discussed in the manuscript. We also note that for scenario 1, the estimated values of the ITR are higher than the value of the optimal ITR. When using finite samples, such results may occur. Increasing the sample sizes of our simulated data sets can help address that issue.
