# Supplementary material for: Model selection for survival individualized treatment rules using the jackknife estimator
Source: BMC Med Res Methodol. 2022 Dec 22;22:328. doi: 10.1186/s12874-022-01811-6 (PMC9773469; doi:10.1186/s12874-022-01811-6)
Supplement: Supplementary file 3 — Additional file 3: Simulations scenarios for assessing the Type I error. [file 12874_2022_1811_MOESM3_ESM.pdf]

Additional file 3: Simulations scenarios for assessing the Type I error

The simulations scenarios for the null model (Type I error) are detailed below.

- **Scenario 1.**

  $\tilde{T}$ is generated from the accelerated failure time model. τ is 1.8 and α is 0.7, 0.32 and -0.05 for 10%, 20% and 40% average censoring rates respectively. Also,

  $\log(\tilde{T}) = -0.2 - 0.5X_1 + 0.5X_2 + 0.4X_3 + 0.15A + \epsilon$, and

  $\log(C) = \alpha - 0.1X_1 + 0.2X_2 + 0.2X_3 + (0.5 - 0.1X_1 - 0.6X_2 + 0.3X_3)A + \xi$

- **Scenario 2.**

  $\tilde{T}$ is generated from the accelerated failure time model with tree-structured effects. τ is 8 and α is 0.5, -0.15 and -1.10 for 10%, 20% and 40% average censoring rates respectively. Also,

  $\log(\tilde{T}) = X_1 + I(X_2 > 0.5)I(X_3 > 0.5) + 2\{I(X_4 < 0.3)I(X_5 < 0.3)\}A + \epsilon$, and

  $\log(C) = \alpha - X_1 + 2X_2 + 2X_3 + (5 - X_1 - 6X_2 + 3X_3)A + \xi$

- **Scenario 3.**

  $\tilde{T}$ is generated from the Cox proportional hazards. τ is 2.5 and α is -0.3, -0.62 and -1.1 for 10%, 20% and 40% average censoring rates respectively. Also,

  $\lambda_{\tilde{T}}(t \mid A, X) = \lambda_0(t) \exp\{-0.2 + 0.75X_1^{1.5} - 0.25X_2$

  $+ 2 * I((1.6 - 1.4X_1^{0.5} - 2.4X_2^2) > 0)A + 0.3 * I((1.6 - 1.4X_1^{0.5} - 2.4X_2^2) < 0)A\}$, and

  $\log(C) = \alpha + 0.5X_1 + X_2 + 0.3X_3 + 0.1X_4 + (0.1 + 0.5X_1 - X_2 + 0.3X_3)A + \xi$.
